# Differentially expressed miRNAs in sepsis-induced acute kidney injury target oxidative stress and mitochondrial dysfunction pathways

**DOI:** 10.1371/journal.pone.0173292

**Published:** 2017-03-15

**Authors:** Qin-Min Ge, Chun-Mei Huang, Xiang-Yang Zhu, Fan Bian, Shu-Ming Pan

**Affiliations:** 1 Department of Emergency, Xinhua Hospital, Shanghai Jiaotong University School of Medicine, Shanghai, China; 2 Division of Nephrology and Hypertension, Department of Internal Medicine, Mayo Clinic College of Medicine, Rochester, MN, United States of America; 3 Department of Nephrology, Xinhua Hospital, Shanghai Jiaotong University School of Medicine, Shanghai, China; University of Cincinnati College of Medicine, UNITED STATES

## Abstract

**Objective:**

To identify specific miRNAs involved in sepsis-induced AKI and to explore their targeting pathways.

**Methods:**

The expression profiles of miRNAs in serum from patients with sepsis-induced AKI (n = 6), sepsis-non AKI (n = 6), and healthy volunteers (n = 3) were investigated by microarray assay and validated by quantitative PCR (qPCR). The targets of the differentially expressed miRNAs were predicted by Target Scan, mirbase and Miranda. Then the significant functions and involvement in signaling pathways of gene ontology (GO) and KEGG pathways were analyzed. Furthermore, eight miRNAs were randomly selected out of the differentially expressed miRNAs for further testing by qPCR.

**Results:**

qPCR analysis confirmed that the expressions levels of hsa-miR-23a-3p, hsa-miR-4456, hsa-miR-142-5p, hsa-miR-22-3p and hsa-miR-191-5p were significantly lower in patients with sepsis compared with the healthy volunteers, while hsa-miR-4270, hsa-miR-4321, hsa-miR-3165 were higher in the sepsis patients. Statistically, miR-4321; miR-4270 were significantly upregulated in the sepsis-induced AKI compared with sepsis-non AKI, while only miR-4321 significantly overexpressed in the sepsis groups compared with control groups. GO analysis showed that biological processes regulated by the predicted target genes included diverse terms. They were related to kidney development, regulation of nitrogen compound metabolic process, regulation of cellular metabolic process, cellular response to oxidative stress, mitochondrial outer membrane permeabilization, etc. Pathway analysis showed that several significant pathways of the predicted target genes related to oxidative stress. miR-4321 was involved in regulating AKT1, mTOR and NOX5 expression while miR-4270 was involved in regulating PPARGC1A, AKT3, NOX5, PIK3C3, WNT1 expression. Function and pathway analysis highlighted the possible involvement of miRNA-deregulated mRNAs in oxidative stress and mitochondrial dysfunction.

**Conclusion:**

This study might help to improve understanding of the relationship between serum miRNAs and sepsis-induced AKI, and laid an important foundation for further identification of the potential mechanisms of sepsis-induced AKI and oxidative stress and mitochondrial dysfunction.

## Introduction

Despite advances in the treatment of critically ill patients, the development of acute kidney injury (AKI) shows a high mortality rate [1]. The mortality rate was 34% in patients with AKI versus 7% in patients without AKI [2]. The cause of AKI in critically ill patients is often multifactorial, while sepsis is the most common cause which is up to 50% [3, 4]. And sepsis-induced AKI is diagnosed in up to 47.9% of ICU patients, it is associated with increased progression to chronic kidney disease (CKD) [1]. Therefore sepsis-induced AKI should be intervened as early as possible. But the mechanisms underlying this event are not fully understood.

There were distinct pathophysiological changes in sepsis-induced AKI compared to non sepsis-induced AKI reflected by special biomarkers, including interleukin-18 (IL-18) whose concentrations were higher in urine of sepsis induced AKI patients [5] Various serum and urinary biomarkers have been found to be up-regulated in kidney injury, such as liver fatty-acid binding protein, cystatin C, kidney injury molecule-1 and neutrophil gelatinase-associated lipocalin [6]. However, they are not specific enough to reflect the pathophysiological changes during early stage of sepsis and AKI.

MicroRNAs (miRNA) are endogenous non-coding RNA molecules which regulate gene expression by degrading mRNA or inhibiting translation. Recent studies demonstrated circulating miRNAs had potential value in diagnosis and prognosis of sepsis [7, 8]. Changes in miRNA expression have been implicated in disease state and reflected the pathophysiological changes during early stage of sepsis when compared with other biomarkers [7, 9]. Circulating miRNAs detected in body fluids are novel biomarkers of renal diseases, including AKI [10, 11]. However, little is known about miRNA expression in septic AKI patients let alone its role in mitochondrial oxidative stress and endothelial dysfunction.

In this study, we profiled serum miRNAs expression levels in sepsis-induced AKI and sepsis-non AKI affected by Gram negative (G^-^) bacteria, identified the differentially expressed miRNAs in sepsis-induced AKI. In addition, we investigated the miRNA expression profiles to confirm the hypothesis that miRNAs alteration in serum might be involved in the mitochondrial oxidative stress and endothelial dysfunction during sepsis-induced AKI.

## Materials and methods

### Patients and blood samples collection

This study was approved by Shanghai Jiaotong University Xinhua Hospital Ethics Committee (XHEC-D-2017-006) and was carried out in accordance with the Declaration of Helsinki. Written informed consents were obtained from all participate in this study. Patients with sepsis were consecutively enrolled from the Xin-Hua Hospital affiliated with the Shanghai Jiaotong University School of Medicine, China, from May 2014 to January 2015. Patients were divided into two groups: the septic-AKI group (meeting the diagnosis of both sepsis and AKI, n = 35) and sepsis-non AKI group (with sepsis but without AKI; n = 30), AKI was defined with KDIGO criteria and sepsis with consensus criteria. Healthy subjects were recruited from the staff of hospital mentioned above. The patients with pre-existing chronic renal failure or chronic use of RRT were excluded from the study. The baseline characteristics and underlying diseases of patients with sepsis-induced AKI, sepsis-non AKI and healthy controls were shown in Table 1. All patients were evaluated with the peak C-reactive protein (CRP), procalcitonin (PCT) and the level of leukocytosis to determine the severity of inflammation. Additionally, the physiological and clinical data were collected, including those that compose the APACHEII (acute physiology and chronic health evaluation II) and SOFA (Sequential Organ Failure Assessment) score. To measure the levels of microRNAs and inflammatory cytokines, serum samples for the 1^st^ day of diagnosis with SIRS or AKI were measured. The bacteria identified by blood culture in the patients with G- were Escherichia coli. There was no statistical significance in terms of age or gender among the groups.

**Table 1:** Baseline characteristics.

**Variables**	**Septic AKI (n = 35)**	**Sepsis-non AKI (n = 30)**	**Control (n = 20)**
Age, years	67.0±10.1	65.3±19.1	55.8±13.2
Female, %(*n*)	65.7(23)	56.7(17)	50(10)
Hypertension, %(*n*)	60.0(21)	30.0(9)	10(2)
DM, %(*n*)	62.9(22)	33.3(10)	5.0(1)
CKD	8.6(3)	0(0)	0(0)

CKD, chronic kidney disease; DM, diabetes mellitus.

### Plasma cytokines assay

Venous blood samples were taken at the time of admission for assessment of serum creatinine concentrations. Venous blood samples were obtained from each patient within 24 h from the first day of diagnosis with sepsis or AKI. The serum was separated, divided in aliquots, and immediately frozen at−80°C until the time of assay. The levels of cytokines including IL-6, IL-8, IL-2sR, IL-10, tumor necrosis factor (TNF)-α and IL-1β were quantified by using an IMMULITE 1000 Automated Immunoassay Analyzer in accordance with the manufacturer’s instructions.

### Microarray analysis of mirna expression profile

15 blood samples, including 6 patients with G- sepsis-induced AKI, 6 patients with G- sepsis-non AKI and 3 healthy controls, randomly selected from the participants in Table 1, After blood collection, we separated serum, isolated total RNA from for miRNA and mRNA analysis. To detect the global miRNA expression profile of sepsis-induced AKI and sepsis-non AKI, we used the TRIzol Reagent (Invitrogen, USA) and a miRNeasy mini kit (Qiagen, Shanghai) to isolate total RNA according to the manufacturers’ instructions. The samples were labeled using the miRCURY™ Hy3™/Hy5™ Power labeling kit (Exiqon, Vedbaek, Denmark) and then hybridized on the miRCURY™ LNA Array (v.18.0) that contained 3100 capture probes. Following the washing steps the slides, the Axon GenePix 4000B microarray scanner (Axon Instruments, Foster City, CA) was used to scan the images. The data was analyzed in GenePix Pro 6.0 software (Axon) for grid alignment and data extraction.

After normalization, significant differentially expressed miRNAs were identified through Volcano Plot filtering between the two experimental groups. Finally, hierarchical clustering was performed to show distinguishable miRNA expression profiling among the samples. The differentially expressed miRNAs were identified by a fold change of ≥1.5.

### Quantitative RT-PCR for miRNA expression

Total RNA was isolated from serum using TRIzol reagent (Invitrogen Life Technologies, USA). The RNA quality and quantity were measured using a NanoDrop ND-1000 spectrophotometer and then quantified using RT-PCR (16°C for 30 min, 42°C for 40 min, and 85°C for 5 min) and a Gene Amp PCR System 9700 (Applied Biosystems). Each RT reaction mixture contained 5 μl Master Mix, 0.5 μl each primer (10 μM), and 2 μl of the corresponding template cDNA, then was incubated at 95°C for 10 min, followed by 40 PCR cycles (95°C for 10 s and then 60°C for 60 s) on a ViiA 7 Real-time PCR System (Applied Biosystems). hsa-miR-423-5p level was used as the endogenous control. The bi-directional primer sequences are presented in S1 Table. The relative expression level for each miRNA was presented by using the comparative cycle threshold (CT) method (2−ΔΔCT).

### Gene ontology (GO) and kyoto encyclopedia of genes and genomes (KEGG) pathway analysis of the deregulated miRNAs

The miRNA target gene database mirbase, miranda and targetscan were used to predict target genes of the differentially expressed miRNAs. Then the final targets were integrated from these three public databases. Using this identified target gene set, we analyzed gene ontology (GO) and KEGG pathway of the significant functions and its involvement in signaling pathways. From the Biological Process, Cellular Component and Molecular Function domains, GO analyze the main function of the predicted target genes and uncover the miRNA-gene regulatory network respectively. Pathway analysis showed that a significant enrichment in several pathways of the predicted target genes. The significance of GO term enrichment and the pathways analysis was defined by *p* value and FDR (a *p*-value<0.05 is recommended).

### Statistical analysis

Data were presented as mean ± SD. The differences in levels of miRNAs among groups were analyzed using two-tailed t-tests or one-way ANOVA. A *P* value <0.05 was considered statistically significant.

## Results

In the present study, 65 patients were diagnosed with sepsis, 53.8% patients developed septic-AKI. The main sources of sepsis were as follows: pulmonary (n = 20; 30.7%), urogenital (n = 15; 23.08%), gastrointestinal (n = 20; 30.7%), and others (n = 10; 15.4%). The patients’ Clinical characteristics and laboratory data were presented in Tables 1 and 2. The mean age of total 65 patients was 66.7±9.9 years and 40 patients (61.5%) were female. The characteristics of the patients were well balanced between the two study groups. Higher level of serum Creatinine, BUN, CRP and PCT were found among the patients with sepsis who developed AKI. The APACHEII score, SOFA score were significantly elevated in patients with septic-AKI compared with those of patients with sepsis but without AKI. However, there were no significant difference in the levels of Bilirubin, Lactate, and 24h urine output as well as 28d in-hospital mortality between the two groups.

**Table 2:** Clinical characteristics of the septic-acute kidney injury (AKI) and the sepsis non-AKI groups.

**Variables**	**Septic AKI (n = 35)**	**Sepsis-nonAKI (n = 30)**	**p**
WBC, 10^9^ cells/L	20.61 ± 12.24	18.41±4.41	0.08
CRP, mg/dL	13.65±5.02	8.92±1.84	0.015
PCT, ng/mL	51.90 ± 39.76	9.56±5.51	0.000
BUN, mg/dL	44.76±7.65	14.02±1.99	0.002
Creatinine, mg/dL	3.24±2.92	0.82±0.20	0.000
APACHEII	19.5±6.8	12.5±4.1	0.001
SOFA	9.8±2.6	6.4±2.8	0.016
Bilirubin, mg/dL	24.8±6.2	14.6±6.1	0.715
Lactate, mg/dL	4.5±2.0	2.4±1.7	0.091
24h urine output (mL)	1086± 500	1059 ± 524	0.897
28d in-hospital mortality, % (n)	17.1(6)	6.7(2)	0.211

Values are presented as mean ± SD.

WBC, white blood cell; CRP, C-reactive protein; PCT, procalcitonin; BUN, blood urea nitrogen.

### Comparison of the level of cytokines in the septic AKI group with those in the sepsis-non AKI and healthy control groups

To further investigate whether inflammation status was different between septic AKI patients and sepsis non-AKI patients, the levels of pro-inflammatory and anti-inflammatory cytokines as well as IL-2sR were studied. As mentioned, the level of cytokines was also compared to exclude the effect of decreased renal excretory function between the septic-AKI and healthy controls groups. In patients with septic AKI, the levels of TNF-α, IL-1β, IL-6, IL-2sR and IL-10 were significantly increased, while the levels of IL-8 was significantly decreased compared with sepsis patients without AKI. There was no statistical significance on the levels of IL-1β, IL-6, IL-2sR and IL-10 in sepsis-non AKI group compared with the control (Table 3).

**Table 3:** Changes of cytokines in plasma in different groups (mean ± SD).

**Cytokines**	**Septic AKI (n = 35)**	**Sepsis-non AKI (n = 30)**	**p**
IL-8, pg/mL	179.67±47.014	740.00±100.851	0.000
IL-2sR, U/ml	2444.67±311.039	947.67±363.148	0.001
TNF-a, pg/mL	57.27±13.155	23.57±5.454	0.002
IL-10, pg/mL	29.87±4.605	7.27±4.450	0.000
IL-1β, pg/mL	13.30±1.852	5.90±1.386	0.001
IL-6, pg/mL	162.10±43.309	12.77±1.629	0.000

### Differential expression of serum miRNAs from the different groups

**Fig 1:**
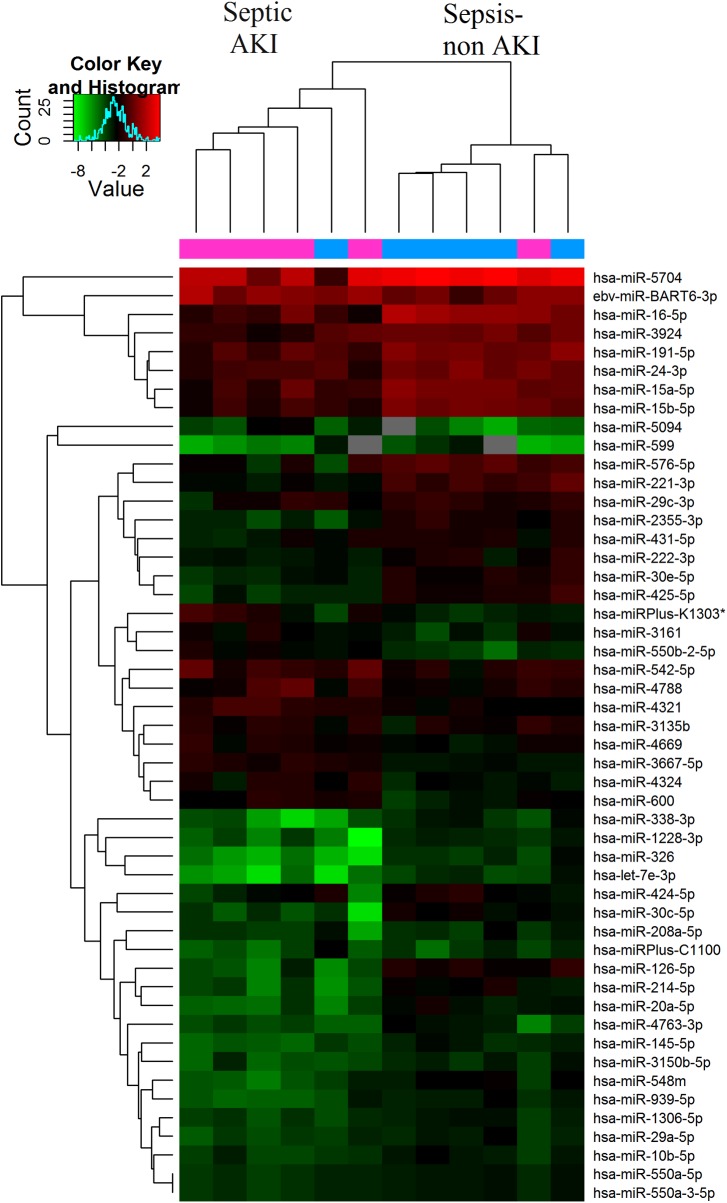
Differentially expressed miRNAs between two groups. Septic AKI (n = 6); Sepsis-non AKI (n = 6). Red color indicated high relative expression and green color denoted low relative expression. miRNA with expression fold change >1.5 and with FDR <0.05 was considered statistically significant.

**Fig 2:**
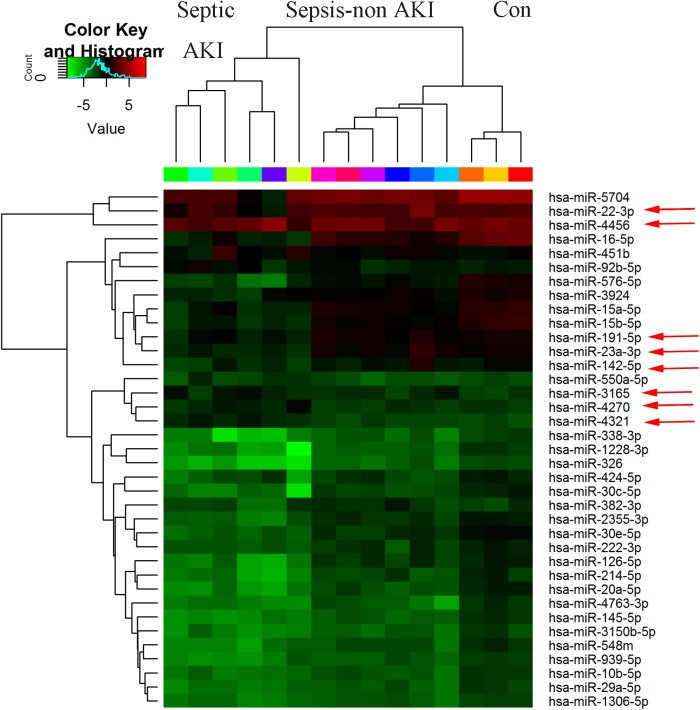
Differentially expressed miRNAs among the three groups. Control (n = 3); Septic AKI (n = 6); Sepsis-non AKI (n = 6). Red color indicated high relative expression and green color denoted low relative expression. miRNA with expression fold change >1.5 and with FDR <0.05 was considered statistically significant. MiRNAs marked with arrow were randomly selected for further confirmation by RT-qPCR.

To identify the most significant candidates, miRNAs with at least 1.5 fold expression changes were selected (S2 Table). A total of 40 miRNAs were differentially expressed between sepsis-induced AKI group and sepsis-non AKI groups (FDR < 0.05). In the sepsis-induced AKI group, 13 of the 40 miRNAs, under the criteria, were up-regulated and 27 miRNAs were down-regulated compared with the sepsis-non AKI group (Fig 1, Tables 4 and 5) Then we profiled the miRNAs among these two groups and the control. A total of 37 miRNAs were differentially expressed among the sepsis-induced AKI group, sepsis-non AKI group and the control (FDR < 0.05) (Fig 2). Among the 37 miRNAs, there were similar expression trends of 8 miRNAs in both the sepsis-induced AKI and sepsis-non AKI group compared with the control. These 8 miRNAs differentially expressed between the sepsis-induced AKI group and the sepsis-non AKI group were hsa-miR-142-5p, hsa-miR-4270, hsa-miR-22-3p, hsa-miR-191-5p, hsa-miR-3165, hsa-miR-4321, hsa-miR-23a-3p and hsa-miR-4456. Three miRNAs (hsa-miR-4270, hsa-miR-4321, hsa-miR-3165) were up-regulated in the sepsis-induced AKI group; and 5 miRNAs (hsa-miR-142-5p, hsa-miR-22-3p, hsa-miR-191-5p, hsa-miR-23a-3p, hsa-miR-4456) were down-regulated.

**Table 4:** Differentially expressed (fold change ≥ 1.5) miRNAs in sepsis-induced AKI and sepsis-non AKI.

Name	Fold change (up-regulated)
hsa-miR-542-5p	1.993055
hsa-miRPlus-K1303*	2.918874
hsa-miR-3667-5p	1.685315
hsa-miR-5094	3.709517
hsa-miR-3135b	1.508861
hsa-miR-550b-2-5p	2.222272
hsa-miR-4669	1.576293
ebv-miR-BART6-3p	1.893911
hsa-miR-4788	2.229297
hsa-miR-4324	1.776153
hsa-miR-4321	1.720649
hsa-miR-600	1.749069
hsa-miR-3161	1.931825

**Table 5:** Differentially expressed (fold change ≥ 1.5) miRNAs in sepsis-induced AKI and sepsis-non AKI.

Name	Fold change (down-regulated)
hsa-miR-24-3p	0.562257
hsa-miR-30e-5p	0.46247
hsa-miR-29a-5p	0.577599
hsa-miR-5704	0.478386
hsa-miR-425-5p	0.428079
hsa-miR-424-5p	0.467525
hsa-miR-3150b-5p	0.55316
hsa-miR-431-5p	0.600788
hsa-miR-4763-3p	0.452261
hsa-miR-326	0.266967
hsa-miR-939-5p	0.512036
hsa-miR-1306-5p	0.6186
hsa-miR-1228-3p	0.489302
hsa-miR-30c-5p	0.425879
hsa-miR-576-5p	0.470405
hsa-miR-126-5p	0.32673
hsa-miR-10b-5p	0.561334
hsa-miR-221-3p	0.44086
hsa-miR-214-5p	0.417281
hsa-miR-550a-3-5p/hsa-miR-550a-5p	0.659555
hsa-miR-2355-3p	0.441743
hsa-miR-208a-5p	0.542032
hsa-miR-222-3p	0.546372
hsa-let-7e-3p	0.336665
hsa-miR-29c-3p	0.652333
hsa-miR-15a-5p	0.475941
hsa-miR-3924	0.490532
hsa-miR-15b-5p	0.489396
hsa-miR-16-5p	0.384366
hsa-miR-548m	0.358639
hsa-miR-145-5p	0.438287
hsa-miR-599	0.201873
hsa-miRPlus-C1100	0.459526
hsa-miR-20a-5p	0.438378
hsa-miR-338-3p	0.376498
hsa-miR-191-5p	0.505111

After normalization, obtained average values for each miRNA spot were used for statistics. MiRNA with expression fold change >1.5 and with FDR <0.05 was considered statistically significant.

**Fig 3:**
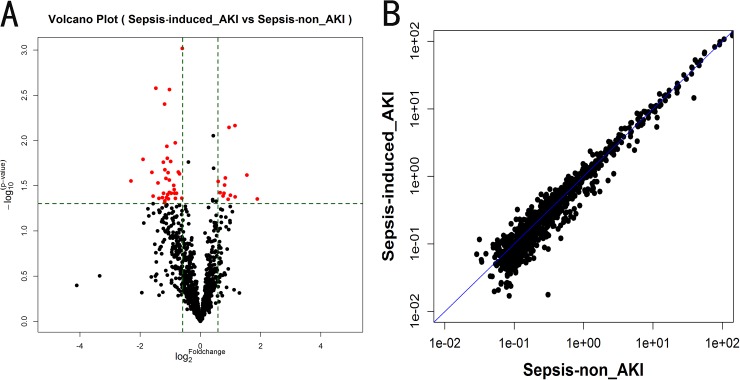
Volcano plot (A) and scatter plot (B) of the miRNA microarray analysis. (A) Volcano plots are useful tool for visualizing differential expression patterns between two different conditions. The vertical lines correspond to 1.5-fold up and down, respectively, and the horizontal line represents a P value of 0.05. So the red point in the plot represents the differentially expressed miRNAs with statistical significance. (B) The scatter plot is a useful visualization for assessing the variation (or reproducibility) between chips. The axes of the scatter plot are the normalized signal values of the samples (the ratio scale).

A scatter plot was generated to assess the quality of the miRNA data after filtering, and a volcano plot was generated to visualize the differential expression between two different conditions, as shown in Fig 3.

### Validation of microarray results by RT-qPCR

Based on the predicted target genes of these miRNAs, we selected eight miRNAs (hsa-miR-4270, hsa-miR-4321, hsa-miR-3165, hsa-miR-23a-3p, hsa-miR-4456, hsa-miR-142-5p, hsa-miR-22-3p, hsa-miR-191-5p) randomly from the differentially expressed miRNAs for further validation.

**Fig 4:**
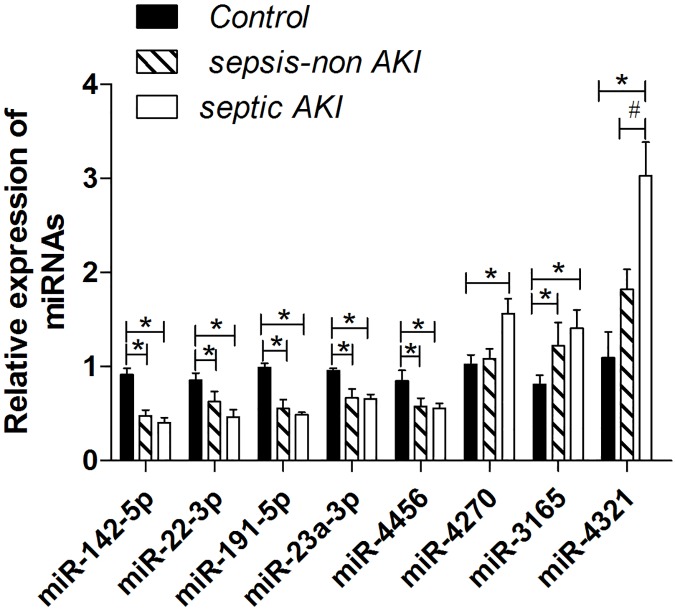
Confirmation of miRNA level by RT-qPCR. Relative expression of each miRNA was normalized to hsa-miR-423-5p level. miR-142-5p, miR-22-3p, miR-191-5p, miR-23a-3p and miR-4456 were significantly decreased in both the sepsis-induced AKI and sepsis-non AKI groups compared with the healthy controls. miR-4270, miR-4321 and miR-3165 were obviously increased in the sepsis-induced AKI, and miR-3165 also was obviously increased in sepsis-non AKI group compared with the healthy controls. While statistical significance between the sepsis-induced AKI and sepsis-non AKI groups was shown only on miR-4321 expression. Note: a single asterisk indicates significant difference between the healthy controls (n = 3) and the sepsis-induced AKI group (n = 6), as well as between the healthy controls and the sepsis-non AKI group (n = 6). A pound sign indicates a statistical difference between the sepsis-induced AKI group and the sepsis-non AKI group. *P* < 0.05 was considered statistically significant.

Relative expression of each miRNA was normalized to hsa-miR-423-5p level. Among the eight selected miRNAs, five miRNAs including hsa-miR-23a-3p, hsa-miR-4456, hsa-miR-142-5p, hsa-miR-22-3p and hsa-miR-191-5p were significantly down-regulated in both the sepsis-induced AKI and sepsis-non AKI groups compared with the healthy controls (*P* < 0.05); three miRNAs including hsa-miR-4270, hsa-miR-4321, hsa-miR-3165 were significantly upregulated in the sepsis-induced AKI group compared with the healthy controls (*P* < 0.05). There was no statistical significance between sepsis-induced AKI and sepsis-non AKI groups on down-regulated miRNAs expression (*P* >0.05). As to the three up-regulated miRNAs, the expression of miR-4270 was up-regulated only in AKI group (*P* < 0.05) with no statistical significance in sepsis-non AKI group compared with the control. The expression of miR-3165 was increased in sepsis groups no matter whether AKI existed or not (*P* < 0.05). While statistical significance among the sepsis-induced AKI, sepsis-non AKI and the control groups was shown only on miR-4321 expression (*P* <0.05) (Fig 4). The miRNAs whose expression levels altered were related to at least 77 mitochondrial oxidative stress and dysfunction-associated target genes based on target predictions (Table 6).

**Table 6:** The differentially expressed miRNAs (sepsis-induced AKI and sepsis-non AKI >1.5) and their predicted target genes.

microRNA	Fold change	Predicted oxidative stress associated target genes
**miR-142-5p**	0.423581	IGF2BP3, PIK3C2A, TNFAIP6, AKT1, IGF1, IL8, IL6, PPARGC1A, SIRT1, NOX5, OXSR1, MTOR, PDCD4, PIK3C2A
**miR-23a-3p**	0.394515	AKT2, PPARGC1A, SIRT1, OXSR1, MTOR, PIK3C2G, IL6R, TNFAIP6, PIK3C2A, FOXO4, PDCD4,IGF1, IL6
**miR-22-3p**	0.563722	AKT3, SIRT1, IL6R, PIK3CD, PPARGC1B, HIF1AN, PIK3CD, IGF1, PDCD2L, PIK3C2G, WNT1
**miR-4456**	0.647923	IGF2BP1, SIRT1, HIF3A, PPARGC1B, IL10RB
**miR-191-5p**	0.505111	OXSR1, FOXO1, PPARGC1A, PIK3C2B, PDCD5, PIK3R3
**miR-4270**	2.333464	AKT3, FOXO4, HIF1A, NOX1, NOX5, PIK3C3, PIK3R1, PPARGC1A, WNT1, PDCD1, IL10RA, IGF2BP1, SIRT5
**miR-3165**	1.500869	IGFBP6, IGFBP7, IL11RA,IL6ST, PPARGC1A, HIF3A, PIK3C2A
**miR-4321**	1.720649	AKT1, MTOR, NOX5, IL17RA, IL26

### GO and KEGG pathway analysis of the deregulated miRNAs

**Fig 5:**
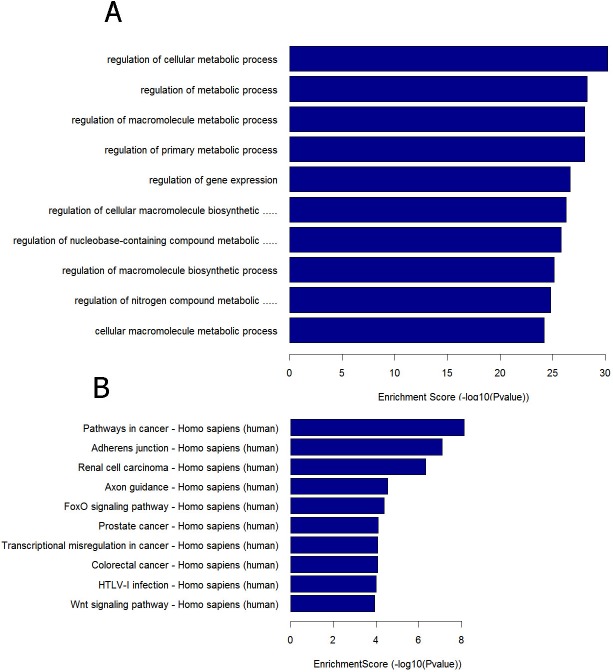
Bioinformatics GO and pathway analysis based on miRNA target genes. Enrichment score is equal to -log10 (P-value) that represents the significant level of GOs and pathways. (A) Significant GOs. (B) Significant signaling pathways. KEGG: Kyoto Encyclopedia of Genes and Genomes; GO: Gene ontology. BH-corrected P < 0.05 was considered statistically significant.

**Fig 6:**
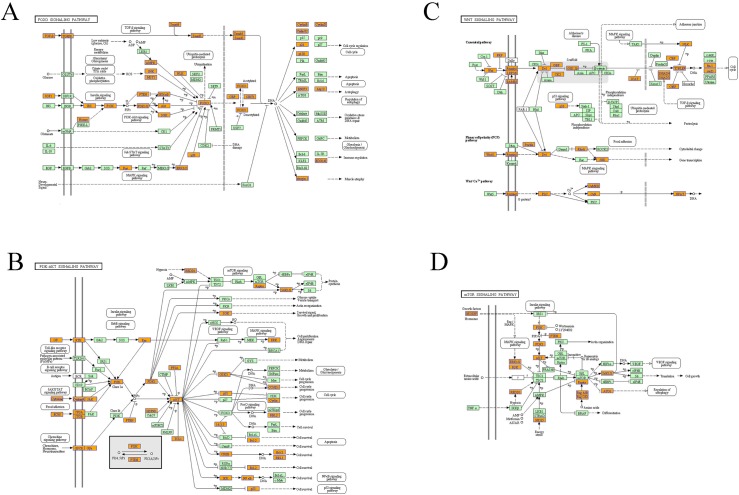
The pathways enriched in the FoxO signaling pathway (A), PI3K-Akt signaling pathway(B), Wnt signaling pathway (C) and mTOR signaling pathway.

**Fig 7:**
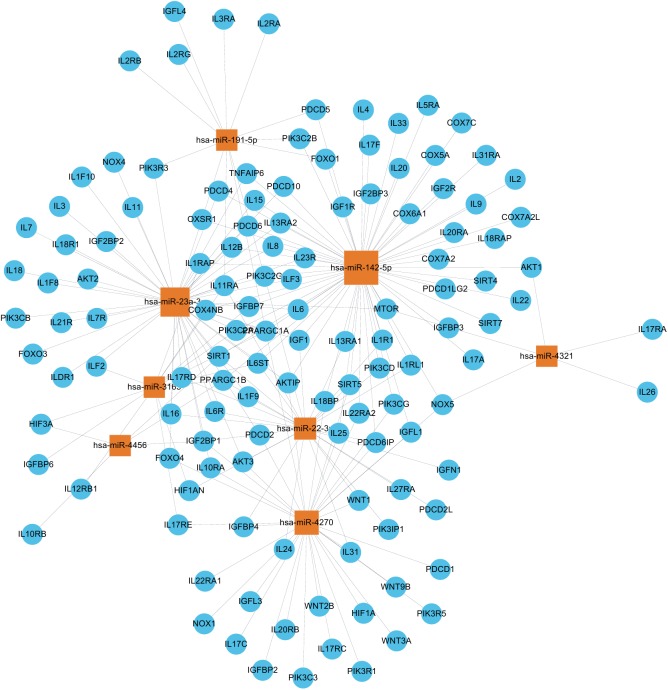
miRNA-mRNA gene network analysis. All the miRNAs in the microarray and GOs were integrated by outlining the interactions of miRNA and GO-related genes

We predicted the potential target genes of these 8 differentially expressed miRNAs among the three groups to further explore the miRNAs function by using Target Scan, mirbase and Miranda. Then the significant functions and involvement in signaling pathways of GO and KEGG pathways were analyzed. GO analysis showed that regulation of cellular metabolic process, regulation of gene expression and regulation of nitrogen compound metabolic process represented the significantly enriched ones. (Fig 5A, S3 Table) Other biological processes regulated by the predicted target genes included kidney development, cellular response to oxidative stress, mitochondrial outer membrane permeabilization and etc. Pathway analysis showed that Wnt signaling pathway and FoxO signaling pathway were abundant among the significantly enriched ones. Other significant pathways of the predicted target genes included HIF-1 signaling pathway, PI3K-Akt signaling pathway, mTOR signaling pathway, TNF signaling pathway, TGF-beta signaling pathway (Fig 5B, S4 Table). They were found to be involved in response to oxidative stress and mitochondrial dysfunction (Fig 6). From the functional analysis of these DE miRNAs using KEGG pathway enrichment analysis, we inferred that circulating miRNAs might participate in septic AKI by influencing immune and inflammatory response indirectly. All target genes of the eight differentially expressed miRNAs in the three databases are shown in S5 Table. In addition, miRNA-mRNA gene network analysis also integrated all the miRNAs in the microarray and GOs by outlining the interactions of miRNA and GO-related genes (Fig 7).

## Discussion

Many recent clinical and experimental studies have shown that sepsis-induced AKI is characterized by endothelial injury with hemodynamic dysfunction [12, 13]. Oxidative stress, a state of reactive oxygen species (ROS) overproduction plays a key role in the pathogenesis of endothelial dysfunction. Release of inflammatory mediators, oxidative stress together with mitochondrial respiratory chain dysfunction can contribute to AKI [14, 15]. Sepsis is the most common cause of acute kidney injury in clinical practice. Inflammation and cytokines play key roles in sepsis pathophysiology [16]. Sepsis-induced AKI was considered as an integrated response, including transcriptional events, ROS signaling, mitochondrial activity and metabolic orientation such as apoptosis [17]. Despite the evidence that mitochondrial oxidative stress and dysfunction are implicated in the development of sepsis induced kidney injury, the precise pathophysiological mechanisms remain unclear.

Emerging evidence demonstrated that miRNAs are intensively engaged in mitochondrial oxidative stress and dysfunction [18]. Present studies demonstrated circulating miRNAs had potential value in diagnosis and prognosis of sepsis. Changes in miRNA expression have been implicated in disease states and reflected the pathophysiological changes during early stage of either sepsis or AKI when compared with other biomarkers. Some serum miRNAs, including miR-21, miR-494, miR-21, miR-155, miR-210, miR-101-1, miR-127-3p, miR-126, miR-26b, miR-29a, miR 146a, miR-27a, miR-93 and miR-10a were identified as the biomarkers for the diagnosis of AKI [19–21], but miRNAs in relation to septic AKI patients have not been previously reported yet.

In this study, we observed that hsa-miR-508-5p was the most significantly up-regulated, whereas, ebv-miR-BART1-3p was the most significantly down-regulated miRNAs in the sepsis-induced AKI group than healthy controls respectively; hsa-miR-508-5p was the most increased and ebv-miR-BART1-3p was the most decreased miRNAs in the sepsis-non AKI group than with the healthy controls respectively; hsa-miR-5094 was the most up-regulated, whereas, hsa-miR-326 was the most down-regulated miRNAs in the sepsis-induced AKI group than the sepsis-non AKI group. Many deregulated miRNAs in our study, for example, miR-222-3p and miR-221-3p have been reported to be involved in specific signaling pathways. miR-222-3p and miR-221-3p were found to promote intracellular ROS accumulation in Human Aortic Endothelial Cells (HAECs) by targeting PGC-1α [22]. However, the biological functions of most differentially expressed serum miRNAs, such as miR-4321 is still largely unknown. In this study, we found that a different subset of miRNAs was dysregulated in sepsis-induced AKI compared to reports of sepsis-non AKI patients. We identified 37 miRNAs differentially expressed among the three groups. While in our work, the majority of the deregulated miRNAs exhibited similar expression trend in sepsis groups compared with the healthy controls, which demonstrated that both sepsis-induced kidney injury and sepsis-non AKI seem to share, at least in part similar regulatory mechanisms. To identify serum miRNAs that might be correlated with resistance or susceptibility to sepsis-induced kidney injury, we focused on the miRNAs differentially expressed between the sepsis-induced kidney injury group and the sepsis-non AKI group. In the present study, we found that 49 miRNAs were differentially expressed among the three groups, of which 13 miRNAs were increased, whereas 36 miRNAs were decreased in the sepsis-induced kidney injury group compared with the sepsis-non AKI group. According to the KEGG pathway and GO analysis regarding target genes of these deregulated miRNAs, we found significantly enriched functions and signaling pathways were associated with oxidative stress and mitochondrial dysfunction. GO analysis showed that regulation of cellular metabolic process, regulation of gene expression and regulation of nitrogen compound metabolic process represented the significantly enriched ones. Other biological processes regulated by the predicted target genes, including kidney development, cellular response to oxidative stress, mitochondrial outer membrane permeabilization, etc. Pathway analysis showed that Wnt signaling pathway and FoxO signaling pathway were abundant among the significantly enriched ones. Other significant pathways of the predicted target genes related to oxidative stress, including HIF-1 signaling pathway, PI3K-Akt signaling pathway, mTOR signaling pathway, TNF signaling pathway, TGF-beta signaling pathway, etc. Function and pathway analysis highlighted the possible involvement of miRNA-deregulated mRNAs in mitochondrial oxidative stress and dysfunction.

To explore the effects of miRNAs on mitochondrial oxidative stress and dysfunction related target gene, we compared the results of miRNA and mRNA. As shown above, an individual miRNA may target multiple mRNAs. In our study, we found that the variation of the 8 miRNA expression were associated with 13 genes which were involved in the activation of mitochondrial oxidative stress and dysfunction response (PPARGC1A (PGC-1α), SIRT1, OXSR1, FOXO1), inflammation (IGF2BP3, IL8, IL6, PDCD4 and TNFAIP6). Notably, 5 miRNAs were involved in PGC-1α regulation; 4 miRNAs were involved in SIRT1 regulation. PGC-1α is a key regulator of mitochondrial biogenesis, the levels of PGC-1α are deregulated when there is damage to the mitochondria during sepsis [23, 24]. SIRT1, upstream of PGC-1α, is also an important modulator of energy metabolism and contributes to the mitochondrial biogenesis program. The SIRT1/PGC-1α axis was involved in mitochondrial biogenesis [25]. A few miRNAs have been found to be associated with mitochondrial oxidative stress and dysfunction in various organs. Overexpression of miR-23a was also found to damage mitochondria in cultured cardiomyocytes, ascribed to PGC-1α downregulation [26]. Overexpression of miR-142 led to a decrease in SIRT1 expression in the brain, and SIRT1 is a direct miR-142-5p target [27]. Our results showed that miR-142-5p, miR-191-5p, miR-3165, miR-4270 and miR-23a-3p were involved in regulating PGC-1α expression; miR-23a-3p, miR-22-3p, miR-4456 were involved in regulating SIRT1 expression. It was worth noting that difference between the sepsis-induced AKI and sepsis-non AKI groups was shown only on miR-4321; miR-4270 was only significantly overexpressed in the sepsis-induced AKI group compared with the sepsis and control groups. Our results indicated that miR-4321 was involved in regulating AKT1, mTOR and NOX5 expression while miR-4270 was involved in regulating PGC-1α, AKT3, NOX5, PIK3C3, WNT1 expression. Acute loss of renal function reduces leukocyte recruitment into inflamed tissue by reducing phosphorylation of Akt and p38 MAPK and transmigration [28]. mTOR is a key kinase downstream of PI3K/AKT, which regulates cell proliferation, growth, survival and angiogenesis. In our sepsis-induced AKI, many of the altered miRNAs were down-regulated which might decrease the expression of PGC-1α directly or indirectly, and weaken the mitochondrial oxidative stress and dysfunction.

Recent evidence from laboratory and clinical studies indicated that AKI in sepsis had a prominent inflammatory component in both the initiation and the extension phases of the kidney injury [29, 30]. The onset of AKI was preceded by early and remarkable inflammatory response and oxidative stress [31]. Inflammatory cytokines, including TNF, interleukin-1β (IL-1β) and IL-6, can induce oxidative stress, stimulate PGC-1α mRNA expression [22]. For a more comprehensive cytokine profile in sepsis, we measured the levels of pro-inflammatory and anti-inflammatory cytokines as well as IL-2sR. Our data showed that pro-inflammatory cytokines, such as TNF-α and IL-8 were significantly increased in the sepsis-non AKI compared with the healthy controls. With the aggravation of the disease, the levels of pro-inflammatory cytokine IL-8 was significantly decreased in the septic AKI compared with sepsis-non AKI group, but still higher than the normal control group; and the levels of pro-inflammatory cytokines TNF-α, IL-1β and IL-6 gradually increased, significantly higher than the normal control and sepsis-non AKI groups; anti-inflammatory cytokines IL-10 was significantly increased in the septic AKI compared with sepsis-non AKI and control group. IL-2sR was significantly increased in patients with septic AKI compared with those of either sepsis-non AKI or healthy controls patients. CD25 is an IL-2 receptor, sCD25 or IL-10 may be useful as a novel biomarker for the development of septic AKI [32]. Several large cohorts of critically ill patients demonstrated that IL-6 could be a robust predictor of AKI [33]. In keeping with our findings, Murugan et al [34] demonstrated that septic AKI patients had higher plasma levels of IL-6 and TNF-α than non-AKI patients. Our study extends the clinical observations by demonstrating the temporal relation between early, exaggerated inflammatory and oxidative stress response and AKI, thus supporting the cause-effect interplay between AKI and abnormal immune response.

To clarify the mechanism of septic AKI occurrence and evaluate the most significant candidates, miRNAs and their possible target genes, which were in the intersection of protein binding and FoxO signaling pathway and Wnt signaling pathway, might be the focus of the further research.

To summarize, we identified specific miRNAs among the sepsis-induced kidney injury group, the sepsis-non AKI group and the healthy controls. The different expression of miRNA might potentially be used to discriminate septic AKI from sepsis-non AKI and they were correlated with the regulation of mitochondrial oxidative stress and dysfunction, including PGC-1α, SIRT1, mTOR, OXSR1 and NOX5. Further study is needed to confirm the relationship between dysregulated miRNAs and their targets, together with the clinical significance of our observations. The interactions between miRNAs and their target genes are complex. The modulation of the oxidative stress and mitochondrial dysfunction by miRNAs is complex as well and requires further investigation.

## Supporting information

S1 Table The bi-directional primer sequences for differentially expressed miRNAs. (XLSX)

S2 Table Differentially expressed circulating miRNAs in septic AKI and sepsis-non AKI group. (XLSX)

S3 Table Functional classification of the target genes by GO analysis. (XLS)

S4 Table Pathway Enrichment analysis. (XLS)

S5 Table All target genes of the eight differentially expressed miRNAs in the three databases. (XLSX)
